# Steered Response Power for Sound Source Localization: a tutorial review

**DOI:** 10.1186/s13636-024-00377-z

**Published:** 2024-11-12

**Authors:** Eric Grinstein, Elisa Tengan, Bilgesu Çakmak, Thomas Dietzen, Leonardo Nunes, Toon van Waterschoot, Mike Brookes, Patrick A. Naylor

**Affiliations:** 1 Department of Electrical and Electronic Engineering, Imperial College London, London, UK; 2 Department of Electrical Engineering (ESAT), STADIUS Center for Dynamical Systems, Signal Processing, and Data Analytics, KU Leuven, Leuven, Belgium; 3 Department of Electrical Engineering (ESAT), Processing Speech and Images, KU Leuven, Sint-Katelijne-Waver, Leuven, Belgium; 4 Microsoft Research, Rio de Janeiro, Brazil

**Keywords:** Sound Source Localization, DOA estimation, Steered Response Power, Acoustic signal processing, Beamforming

## Abstract

In the last three decades, the Steered Response Power (SRP) method has been widely used for the task of Sound Source Localization (SSL), due to its satisfactory localization performance on moderately reverberant and noisy scenarios. Many works have analysed and extended the original SRP method to reduce its computational cost, to allow it to locate multiple sources, or to improve its performance in adverse environments. In this work, we review over 200 papers on the SRP method and its variants, with emphasis on the SRP-PHAT method. We also present eXtensible-SRP, or X-SRP, a generalized and modularized version of the SRP algorithm which allows the reviewed extensions to be implemented. We provide a Python implementation of the algorithm which includes selected extensions from the literature.

## Introduction

Sound Source Localization (SSL) is the task of estimating the position of one or more active acoustic sources using one or more microphone arrays. Applications for SSL include event detection [1–3], camera steering [4], and sound source separation [5–7] among many others. In the last decades, many classical signal processing-based methods were developed for SSL, including Multiple Signal Classification (MUSIC) [8], Estimation of Signal Parameters via Rotational Invariance Techniques (ESPRIT) [9], Time-Difference-of-Arrival (TDOA)-based [10, 11], Maximum Likelihood (ML)-based [12] and Steered Response Power (SRP) [13, 14], which is the focus of this review. Alternatively to signal processing-based methods, significant research interest has also been devoted to machine learning-based localization methods [15].

Choosing a localization method from all the available methods depends on the type of available acoustic and computational resources, assumptions about the localization scene, and knowledge of the method’s mathematical formulation. SRP is known for its straightforward formulation and robust performance in many realistic environments [16]. A historical disadvantage of the method has been its significant computational complexity, although this is of diminishing importance due to the increased computational capacity of today’s devices and to the many optimized modifications of SRP which have been developed. This has resulted in SRP becoming a standard SSL method in the literature.

Besides reducing its computational complexity, dozens of SRP variants have been developed to improve aspects of its performance, including increasing its robustness in adverse environments or in specific scenarios, and allowing multiple sources or moving sources to be localized. SRP can also be used as a feature extractor for neural-based localizers [17]. Therefore, one must not only choose SRP as a localizer, but must also decide which of the multiple SRP ‘flavours’ to use. A prominent flavour is the SRP-PHAT method, which uses the Generalized Cross-Correlation with Phase Transform (GCC-PHAT) [18] method as its correlation function, which is shown to offer advantages to other correlation functions for processing speech signals. Unless stated otherwise, the term SRP refers to SRP-PHAT throughout this work.

Although many reviews on the broader field of SSL exist in the literature [15, 19, 20], no work has exclusively focused on SRP and its variants which allow it to be applied in practice. The goal of this paper is therefore to provide a centralized resource for SRP research, to be used by both newcomers and experienced practitioners in the field of SSL. Over 200 papers are classified, described and compared, followed by the developement of a modular description of the algorithm, which can be used to develop implementations. A code library named X-SRP is also released as part of this work, with the goal of facilitating the usage of the algorithm. The remainder of the paper comprises the following sections:

2. *The conventional SRP model*, which presents SRP along with the relevant acoustics concepts required for its comprehension.
3. *Reducing SRP’s complexity and computational time*, which discusses papers that focus on reducing SRP’s computational cost at a minimal decrease in localization performance.
4. *Increasing robustness*, which focuses on improving SRP’s performance on reverberant and noisy environments using, for example, neural network methods.
5. *Multi-source SRP approaches*, which generalizes the conventional SRP definition to the detection and localization of multiple simultaneously active sound sources.
6. *Practical considerations*, which include practical applications involving SRP, adaptations of the method to track moving sources, to exploit and estimate source and microphone directivity, and comparisons to alternative SSL methods.
7. *X-SRP*, where a modular description of SRP is provided by decomposing the algorithm into functional building blocks. Each of the reviewed papers usually modify a single block in the proposed framework, allowing works to be combined and altered. We apply the created framework by releasing an open-source Python implementation of SRP denoted X-SRP, or eXtensible-SRP, with the goal of facilitating collaboration in the field. The released code^1^ includes implementations of many popular SRP variants.
8. *Conclusion*, where a discussion of future research directions is provided and the work is concluded.

## The conventional SRP model

The earliest descriptions of SRP were provided by Omologo et al. [13, 21, 22] and Dibiase et al. [14, 23]. Earlier works on SRP have also referred to the method as Global Coherence Field (GCF) [24–28]. The method was later generalized as a Spatial Likelihood Function (SLF) [29]. The term SRP comes from its guiding principle of searching, or *steering* towards a location which maximizes the output power of a beamformer applied to the microphone signals. Alternatively, SRP can also be defined for each pair of microphone signals as the projection of their cross-correlation function in space. Due to its increased clarity, the latter formulation is adopted in this paper.

This section starts by defining the scope of the problem, followed by the signal model used throughout this paper. Finally, a description of SRP’s base model as presented in [14, 23] is provided. Two alternative formulations are presented, the first in the time domain and the second in the frequency domain, as both are commonly encountered in the literature.

### Problem statement and definitions

The goal of a localization method is to estimate the positions of one or more sound sources located in space, often an indoor environment. This section focuses on the scenario where a single, static and omnidirectional source located at $\textbf{u} = [u^{(1)} \, u^{(2)} \, u^{(3)}]^T$ emits a signal *s*(*t*) at time *t*; the case of directive, moving and multiple sources are respectively discussed in Sections 6.3, 6.2 and 5. The source can also be expressed in spherical coordinates $\textbf{u} = [\phi \, \theta \, \rho ]^T$ with respect to a reference point, typically the centre of a microphone array. Variables $\phi$, $\theta$ and $\rho$ respectively represent the source’s *azimuth*, *elevation* and *range*. The source locations are estimated using signals $x_m(t)$ received from an array of *M* microphones, each located at known positions $\textbf{v}_m = [v_m^{(1)} \, v_m^{(2)} \, v_m^{(3)}]^T$, $m=1,\,... ,\,M$.

#### Near- versus far-field localization

This subsection discusses the different types of localization which are frequently encountered in the literature, namely, Positional Sound Source Localization (PSSL) and Direction-of-Arrival (DOA) estimation. PSSL consists of fully estimating the source’s position and is usually employed when the distances between microphones in the array is similar to the distance between the microphones and the source. This is equivalent to saying the source is located in the *near-field* of the array. This configuration is referred to as a *distributed* array, which can be constituted for example of multiple network-connected devices such as laptops, cell phones or voice assistants. In this case, as each device has their own Analogue-to-Digital Converter (ADC), they must be synchronized to a common sampling frequency $f_s$, or a compensation algorithm must be applied to the signals to prevent synchronization issues [30].

Conversely, when employing a centralized microphone array such as a single voice assistant, the distance between microphones is usually significantly smaller than the distance between the sources of interest and the array itself. This is equivalent to saying the source is located in the array’s *far-field*. In this case, the spherical wave leaving the source is observed as a plane wave which has no defined origin: an infinite set of sources may produce a plane wave with the same incident angle to the array. For this reason, the range $\rho$ is usually not estimated when using compact arrays. The task of estimating the azimuth, $\phi$, and elevation, $\theta$, is referred to as DOA estimation.

### Signal model

The received signal $x_m(t)$ at microphone *m* is equal to

1 $$\begin{aligned} x_m(t) = \int _{-\infty }^{\infty } h_m(r; \textbf{u}) s(t - r) \textrm{d}r + \epsilon _m(t), \end{aligned}$$

that is, a convolution between the source signal *s*(*t*) and a Room Impulse Response (RIR) $h_m(r; \textbf{u})$, which models the propagation effects and reverberation, plus a noise term $\epsilon _m(t)$. However, SRP adopts a simplified propagation model where reverberation is modelled using the noise term $\epsilon _m(t)$. This free-field is defined as

2 $$\begin{aligned} x_m(t) = a_m(\textbf{u}) s(t - \tau _m(\textbf{u})) + \epsilon _m(t) , \end{aligned}$$

that is, the signal emitted by the source is received at microphone *m* attenuated by a factor $a_m(\textbf{u})$, delayed by $\tau _m(\textbf{u})$ seconds and corrupted by a measurement noise term $\epsilon _m(t)$. This is equivalent to adopting the RIR in (1) as a pure impulse $h_m(t; \textbf{u}) = a_m(\textbf{u}) \delta (t - \tau _m(\textbf{u}))$. Note that this model assumes attenuation to be frequency-independent. The attenuation and delay effects will be further detailed in Section 2.3.

Alternatively, it is often advantageous to define (2) in the time-frequency domain, by decomposing the source signal into complex-valued sinusoids $\bar{s}(t, f)$ of frequencies *f*. In practice, such a signal can be obtained by applying the Fourier transform on *s*(*t*). The received signal $\bar{x}_m(t, f)$ is then defined for each time-frequency pair (*t*, *f*) as

3 $$\begin{aligned} \bar{x}_m(t, f) = \bar{s}(t, f)a_m(\textbf{u}, f)e^{-jf\tau _m(\textbf{u})} + \epsilon _m(t, f). \end{aligned}$$

The advantage of (3) in comparison to (2) is that delay, $\tau _m$, and attenuation, $a_m(f)$, effects can be jointly represented by multiplication with a signal complex-valued scalar.

Although the above definitions are conceptually useful, in practice, SRP is computed using a *frame* or vector of dimension *L* samples for each microphone. A frame $\textbf{x}_m(t)$ is defined in the time domain as

4 $$\begin{aligned} \textbf{x}_m(t) = [x_m(t) \, x_m(t-T_s) \, ... \, x_m(t-(L-1)T_s)]^T, \end{aligned}$$

where $T_s = 1/f_s$. Furthermore, a frequency domain frame $\bar{\textbf{x}}_m(t)$ is defined as

5 $$\begin{aligned} \bar{\textbf{x}}_m(t) = \text {DFT}(\textbf{x}_m(t)), \end{aligned}$$

that is, the application of the Discrete Fourier Transform (DFT) to temporal frame $\textbf{x}_m(t)$. $\bar{\textbf{x}}_m(t)$, where each of its entries represents a time-frequency bin $\bar{x}_m(t,f)$ with $f \in \mathcal {F}$, where

6 $$\begin{aligned} \mathcal {F} = \{f| f=-f_s/2 + kf_s/L, \, k=0,...,L-1\}, \end{aligned}$$

constitutes the set of analysis frequency components used.

### Acoustics, TOF and TDOA

In this subsection, we further contextualize the signal model defined in (2) and (3) using relevant acoustic principles.

A sound wave emanating from the source location $\textbf{u}$ travels at the speed of sound *c* to each microphone’s location $\textbf{v}_m$. The propagation time $\tau _m(\textbf{u})$, also known as the Time-of-Flight (TOF) between the source at $\textbf{u}$ and microphone *m*, can therefore be expressed, in seconds, as

7 $$\begin{aligned} \tau _m(\textbf{u}) = \frac{\Vert \textbf{u} - \textbf{v}_m \Vert }{c}. \end{aligned}$$

If $\tau _m(\textbf{u})$ can be correctly estimated for three or more microphones, an estimate of $\textbf{u}$ can be obtained. This is the strategy used by *active* localization systems [12], which use controlled and/or known source signals so that the emission time of the source signal is accessible. Conversely, SRP is a *passive* localization method which allows for a broader range of sources, such as human speakers, to be localized.

SRP performs passive localization by exploiting the *relative* delay, also known as the Time-Difference-of-Arrival (TDOA), between *pairs* of microphones. The importance of the TDOA and its relationship with the cross-correlation function between pairs of signals will be discussed in detail in Section 2.4. Using (7), $\tau _{lm}$ is defined, in seconds, as

8 $$\begin{aligned} \tau _{lm}(\textbf{u}) = \tau _l(\textbf{u}) - \tau _m(\textbf{u}) = \frac{\Vert \textbf{u} - \textbf{v}_l \Vert - \Vert \textbf{u} - \textbf{v}_m \Vert }{c}. \end{aligned}$$

**Fig. 1:**
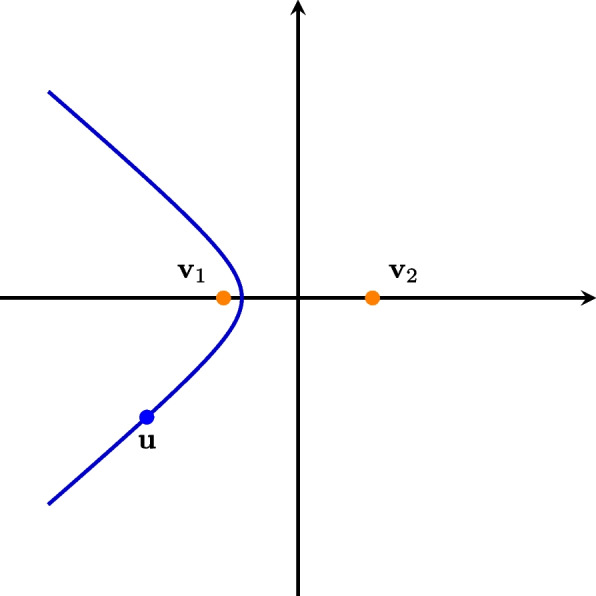
Hyperbola branch of points with the same TDOA as a source located at $\textbf{u}$ with respect to microphone positions $\textbf{v}_1$ and $\textbf{v}_2$. The axes represent the horizontal directions, in meters

The TDOA for a pair of microphones can be interpreted as how much earlier/later a signal arrives at the first microphone in comparison to the Time-of-Arrival (TOA) at the second microphone. Multiple positions $\textbf{u}$ can produce the same delay $\tau _{lm}$ for a pair of microphones fixed at $(\textbf{v}_l, \textbf{v}_m)$. These positions lie along a hyperbola/hyperboloid branch in 2D/3D, as shown in [11] and can be viewed in Fig. 1.

In the case of compact arrays where the near-field assumption holds (Section 2.1.1), the TDOA definition in (8) can be approximated as

9 $$\begin{aligned} \tilde{\tau }_{lm}(\textbf{u}) = \frac{(\textbf{v}_l - \textbf{v}_m)^T}{c} . \frac{\textbf{u}}{\Vert \textbf{u} \Vert }, \end{aligned}$$

that is, the dot product between the vector $\textbf{v}_l - \textbf{v}_m$ and the normalized source direction $\frac{\textbf{u}}{\Vert \textbf{u} \Vert }$, scaled by 1/*c*.

The maximum possible TDOAs for a microphone pair occurs when the source and microphones are collinear and the source is not located between the microphones, and has an absolute value of

10 $$\begin{aligned} |\tau _{lm}^{\text {lim}} |= \frac{\Vert \textbf{v}_l - \textbf{v}_m \Vert }{c}. \end{aligned}$$

By determining the intersection of the hyperbolas produced by multiple microphone pairs, the source position, can be estimated as $\hat{\textbf{u}}$. Approaches utilizing this strategy are known as *triangulation*, *TDOA-based*, *indirect* or *two-step* approaches [12], since they require a first step of estimating the TDOAs before a second step of estimating the source locations. Although these approaches are less computationally expensive than SRP, their reliance on the estimated TDOAs make them non-robust in adverse noisy or reverberant scenarios [31].

### Estimating TDOA: cross-correlation and GCC-PHAT

The TDOA $\tau _{lm}$ between two microphones can be estimated as the argument of the peak of the cross-correlation between microphone signal frames $\textbf{x}_l(t)$ and $\textbf{x}_m(t)$. The discrete cross-correlation (CC) function is defined as

11 $$\begin{aligned} \text {CC}( \tau ; \textbf{x}_l, \textbf{x}_m) = \textbf{x}_l^T(t)\textbf{x}_m(t-\tau ), \end{aligned}$$

where $\tau$ must be a multiple of the sampling period $T_s$ and appropriate zero padding is applied.

Despite its straightforward formulation, (11) is seldom used in practice for localizing speech sources in reverberant and noisy environments, as the non-flat spectrum of the source signal reduces the selectivity of the function. Instead, the Generalized Cross-Correlation with Phase Transform (GCC-PHAT) function [18, 22] is usually adopted, as it was shown to be a better feature for localizing speech sources. ‘Generalized’ comes from the fact that a cross-correlation value is produced for every frequency component of the signals after a pre-filtering operation. This operation is typically the ‘Phase Transform’ weighting, which whitens the frequency components, thus sharpening the correlation peak. The importance of sharp peaks will be made clearer in Section 2.5. The GCC-PHAT function is defined for each time-frequency bin as

12 $$\begin{aligned} \text {GCC-PHAT}(f ; \bar{\textbf{x}}_l, \bar{\textbf{x}}_m) = \frac{\bar{x}_l(t,f)\bar{x}^{*}_m(t,f)}{\left| \bar{x}_l(t,f)\right| \left| \bar{x}_m(t,f) \right| }. \end{aligned}$$

**Fig. 2:**
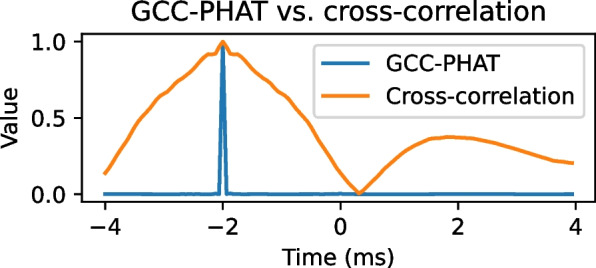
Example comparison between the normalized temporal cross-correlation and GCC-PHAT for a scenario containing two microphones and a source producing a speech signal with a TDOA of −2 ms

The phase transform is applied using the denominator of (12). In practice, (12) is computed for a set of analysis frequencies $\mathcal {F}$ to generate a GCC frame $\bar{\textbf{g}}$. In practice, the Fast Fourier Transform (FFT) algorithm of size *L* is used, where *L* is typically chosen to be a power of 2, to obtain their frequency-domain representation. In those cases, $\mathcal {F}$ is thus implicitly defined as $\lfloor (L/2) \rfloor + 1$ uniformly spaced non-negative frequencies up to the Nyquist rate, where $\lfloor \cdot \rfloor$ represents the floor operation. Conversely, by applying the Inverse DFT (IDFT) to $\bar{\textbf{g}}$, a time-domain vector $\textbf{g}$ can obtained,

13 $$\begin{aligned} \textbf{g} = \text {IDFT}(\bar{\textbf{g}}), \end{aligned}$$

where each entry $\textbf{g}[k]$ represents a temporal correlation value between $\textbf{x}_l$ and $\textbf{x}_m$ at sample *k*. A frame can be built in a similar manner using the temporal CC in (11). The magnitude normalization in (12) improves the resolution of (13) by giving equal weight to all frequency components and focusing on phase information only. An example comparison between two frames computed using (13) and temporal $\text {CC}$ is shown in Fig. 2, where it can be observed that the peak produced by GCC-PHAT is much sharper than by $\text {CC}$. This can be explained because the PHAT operation makes the source signal white, which results in a single peak in its autocorrelation function.

In an ideal scenario, the temporal CC or GCC-PHAT function exhibits a sharp peak at $\tau _{lm}$, which can be used for two-step methods. However, under reverberant or noisy scenarios, the cross-correlation function can exhibit multiple peaks which are higher than those related to the true source position, rendering the TDOA estimates and the subsequent triangulation-based approaches unreliable. This is particularly detrimental when performing multi-source localization, which will be discussed in Section 5, as two peaks of different levels must be detected. As we will show in the following section, SRP mitigates this issua by applying the principle of least commitment [31, 32]; instead of estimating $\tau _{lm}$ early on and discarding all other values and peaks of the cross-correlation function, SRP associates each cross-correlation value with a candidate locus in space using (8).

### Time-domain SRP formulation

The conventional SRP for a candidate source location $\textbf{u}$ and a pair of microphones (*l*, *m*) is defined as [14, 23]

14 $$\begin{aligned} \text {SRP}_{lm}(\textbf{u} \, ; \,\, \textbf{x}_l, \textbf{x}_m) = \text {CC}(\lfloor \tau _{lm}( \textbf{u}) \rceil ; \textbf{x}_l, \textbf{x}_m), \end{aligned}$$

that is, the cross-correlation function between signal frames $\textbf{x}_l$ and $\textbf{x}_m$, evaluated at delay $\lfloor \tau _{lm}( \textbf{u}) \rceil$, where $\lfloor \cdot \rceil$ represents rounding to the nearest multiple of $T_s$. Finally, the global SRP is defined as the sum of all pairwise SRPs,

15 $$\begin{aligned} \text {SRP}(\textbf{u} \, ; \,\, \mathcal {X}) = \sum \limits _{l=1}^M \sum \limits _{m=l+1}^{M} \text {SRP}_{lm}(\textbf{u} \, ; \,\, \textbf{x}_l, \textbf{x}_m), \end{aligned}$$

where $\mathcal {X} = \{\textbf{x}_1,\, ...,\, \textbf{x}_M \}$ is the set of *M* *L*-dimensional frames pertaining all microphones. This value is related to the likelihood of a source being located at a candidate point $\textbf{u}$. The complete SRP method consists of evaluating (15) for a set of candidate locations and selecting the location maximizing (15) as the estimated location. Note that time-domain $\text {GCC-PHAT}$ defined in (13) is usually preferred to (11), as the latter’s high correlation values in the neighbouring delays may cause the source position’s neighbours to exhibit a higher value than the true location. The set of candidate locations typically consists of a regularly sampled spatial grid. The grid construction procedure will be defined in Section 2.7.

### Frequency-domain SRP formulation

This formulation decomposes the microphone signals into frequency bands, which are independently analysed using GCC-PHAT in (12) as

16 $$\begin{aligned} \overline{\text {SRP}}_{lm}( \textbf{u}, f \, ; \,\, \bar{\textbf{x}}_l, \bar{\textbf{x}}_m) = & \nonumber \\ & \text {Re}[\text {GCC-PHAT}(f ; \bar{\textbf{x}}_l, \bar{\textbf{x}}_m) e^{jf\tau _{lm}(\textbf{u})}]. \end{aligned}$$

Equation (16) can be interpreted as steering, or shifting, the microphone signal $x_m(f)$ by a phase $f\tau _{lm}(\textbf{u})$. The extraction of the real part $\text {Re}(\cdot )$ has the goal of measuring the power of signal $x_m(f)$ at position $\textbf{u}$ [14]. Finally, the global $\overline{\text {SRP}}$ is represented in the frequency domain in a similar way to the time-domain formulation (15), after summing over the set $\mathcal {F}$ of frequencies being analysed,

17 $$\begin{aligned} \overline{\text {SRP}}(\textbf{u} \, ; \,\, \bar{\mathcal {X}}) = \sum \limits _{l=1}^M \sum \limits _{m=l+1}^{M} \sum \limits _{f \in \mathcal {F}} \text {SRP}_{lm}(\textbf{u}, f \, ; \,\, \bar{\textbf{x}}_l, \bar{\textbf{x}}_m), \end{aligned}$$

where $\bar{\mathcal {X}}$ is the frequency-domain representation of $\mathcal {X}$. If information on the noise conditions of the signals being analysed is known a priori, frequency bins with high noise content can potentially be discarded from $\mathcal {F}$. Furthermore, the frequencies $\mathcal {F}$ used can also be limited up to the (spatial) Nyquist rate to prevent a phenomenon called spatial aliasing [32, 33], in which the phase ambiguity of certain frequency components may cause localization ambiguity when evaluating the SRP function over the selected set of candidate locations [34]. We note that such phase ambiguity depends on the relationship between the microphone array configuration, specifically the distances between microphone pairs, and the incident angle of the signal wavefront relative to each pair.

Note that the time and frequency definitions of SRP are not equivalent due to this frequency-domain filtering, as well as the rounding operator required when constructing a temporal CC or GCC vector makes (15) operate using integer delays, which may not correspond to the true source’s TDOA. The error due to rounding may be reduced by using distributed arrays or mitigated by applying interpolation [35, 36]. However, significant errors may be produced for compact arrays, where the TDOA range defined by (10) is typically only a few samples [37].

### Grid construction and search

To estimate the location of the source, (15) or (17) are evaluated over a set, $\mathcal {G}$, of *G* candidate positions relative to a reference point in the room, typically one of its corners. The elements of $\mathcal {G}$ are usually defined by creating a uniform spatial grid. For performing SSL in a cuboid-shaped room, a cuboid-shaped grid is typically used. For example, when performing planar or 2D localization $G = G^{(1)} G^{(2)}$, where $G^{(1)}$ and $G^{(2)}$ are respectively the number of points used for the width and length dimension. $\mathcal {G}$ becomes

18 $$\begin{aligned} \mathcal {G}_{2D} = & \; \{ \; [g^{(1)} R^{(1)} \,\, g^{(2)} R^{(2)}]^T \mid \nonumber \\ & g^{(1)} \in \{1,\ldots ,G^{(1)}\} \nonumber \\ & g^{(2)} \in \{1,\ldots , G^{(2)} \} \}, \end{aligned}$$

where $R^{(1)} = D^{(1)}/G^{(1)}$ and $R^{(2)} = D^{(2)}/G^{(2)}$ are the width and length *resolution* for a room of width $D^{(1)}$ and length $D^{(2)}$. Conversely, when performing planar or 2D DOA estimation, the grid can be made by setting the origin to the microphone array centre, and a circular grid is created,

19 $$\begin{aligned} \mathcal {G}_\text {DOA2D} & = \nonumber \\ & \{ \; [\cos (\phi ) \,\, \sin (\phi ])]^T \mid \phi \in \nonumber \\ & \{R^{(\phi )}, 2R^{(\phi )} \ldots , 2\pi \} \}. \end{aligned}$$

In (19), each point represents a distinct candidate source direction. Furthermore, neighbouring points are separated by the angular resolution $R^{(\phi )}$, where $\phi$ is the candidate source’s *azimuth*. In 3D DOA estimation, the *elevation*, defined as the angle between the segment connecting the source and array centre and the horizontal plane is also estimated.

For both DOA estimation and PSSL, the complete SRP map consists of evaluating the SRP function for all candidate locations in the grid $\mathcal {G}$, and selecting the location producing the maximum SRP value as the estimated position,

20 $$\begin{aligned} \hat{\textbf{u}} = \underset{\textbf{u} \in \mathcal {G}}{\text {arg max}}\ \text {SRP}(\textbf{u}). \end{aligned}$$

**Fig. 3:**
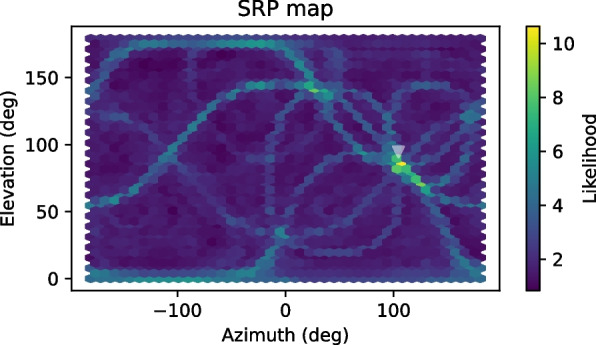
Example of an SRP map for the task of 3D DOA estimation of a speech source using a spherical array of 8 microphones. Reverberation was simulated with a reverberation time of $T_{60} = 400$ ms, and the source is located below the transparent triangle at $(100^o, 60^o)$. Spatially uncorrelated white noise was added to the microphones at 20 dB SNR

An example of an SRP map for a simulated environment of low reverberation is shown in Fig. 3.

## Reducing SRP’s complexity and computational time

### Complexity analysis

We start by outlining the computational complexity of the frequency-domain, conventional SRP method as defined in (17). Here, complexity is measured by the number of real multiplications and divisions performed by the algorithm, ignoring the additions, as commonly done. Furthermore, we follow the Bachmann-Landau (or big-*O*) notation, which measures asymptotic behaviour of algorithm complexity as input sizes grow.

The method can be divided into four sequential operations. The first two operations consist of extracting the DFT for each frame of the *M* microphones followed by computing the GCC-PHAT for all *P* microphone pairs, where $P = M(M-1)/2$. In practice, the FFT algorithm [38] is used to implement the DFT. The FFT has a complexity of $\text {O}(L \log L)$ . We assume the FFT operation converts a time-domain frame of size *L* into a frequency-domain frame of same size. Since GCC-PHAT consists of an element-wise multiplication of the vectors $\bar{\textbf{x}}_l$ and $\bar{\textbf{x}}_m$ divided by their respective magnitudes, its complexity is therefore $\text {O}(L)$.

The third step is the creation of the *P* pairwise SRP likelihood grids of size $G=|\mathcal {G} |$, for all *L* frequencies, followed by their sum to create a global SRP grid. As this operation consists of multiplying the GCC-PHATs by an exponential $e^{jf\tau _{lm}(\textbf{u})}$, its complexity is $\text {O}(GPL)$. The final step consists of comparing all grid points to obtain the argument of its maximum, which is the estimated source location. As comparisons are often assumed to offer a lower complexity, this last step is ignored. The number of operations performed by SRP is thus obtained as

21 $$\begin{aligned} \text {O}_{\overline{\text {SRP}}} & = \text {O}\left( M L \log L + PL + GPL \right) , \nonumber \\ & \simeq \text {O}\left( M L \log L + GPL \right) , \end{aligned}$$

where the three terms in the first line represent each of the sequential operations discussed above. The simplification on the bottom line is obtained by removing the second term, as $G \gg 1$. We can see from (21) that straightforward strategies can be followed to reduce the complexity of SRP. One is to use only a subset of microphones $M' < M$ or subselecting $P' < M(M - 1)/2$ pairs instead of evaluating all pair combinations. Another is to employ a smaller frame size *L* and reducing the frequency range in which the SRP map is computed. Finally, a coarser grid can be employed. All these strategies come, however, with a reduction in localization performance. Most of the research presented in this section proposes strategies to reduce the grid size *G*, or modify the functionality of the conventional SRP method while minimizing the loss in localization performance.

In turn, the computational complexity of time-domain SRP in (15) is smaller than in (21), as a single map is computed in the time domain instead of *L* frequency domain maps, i.e. it uses one less nested ‘for each’ loop. The complexity of (15) is therefore expressed as

22 $$\begin{aligned} \text {O}_\text {SRP} = \text {O}\left( (M+P) L \log L + PL + GP \right) , \end{aligned}$$

where the inverse DFT used to obtain the temporal GCC vector (13) has complexity $L \log L$ and needs to be computed for all P microphone pairs. Furthermore, projection of the cross-correlation function is achieved in (15) by accessing an element in the cross-correlation vector, which is more computationally efficient, albeit less precise, than the multiplication by a complex exponential used in the frequency-domain version.

### Coarse grids and Volumetric-SRP

As mentioned above, reducing *G* is a straightforward strategy for reducing SRP’s complexity. When applying equispaced grids such as those described in (18) and (19), this can be achieved by reducing the resolution parameters $R^{(1)}$, $R^{(2)}$ and $R^{(\phi )}$. However, this comes with the risk of not sampling the true source location, which may lead to the peak of the cross-correlation function not to be projected into the map, leading to a high localization error [39]. Nonetheless, many strategies can be applied to increase the localization performance of approaches using coarse grids.

**Fig. 4:**
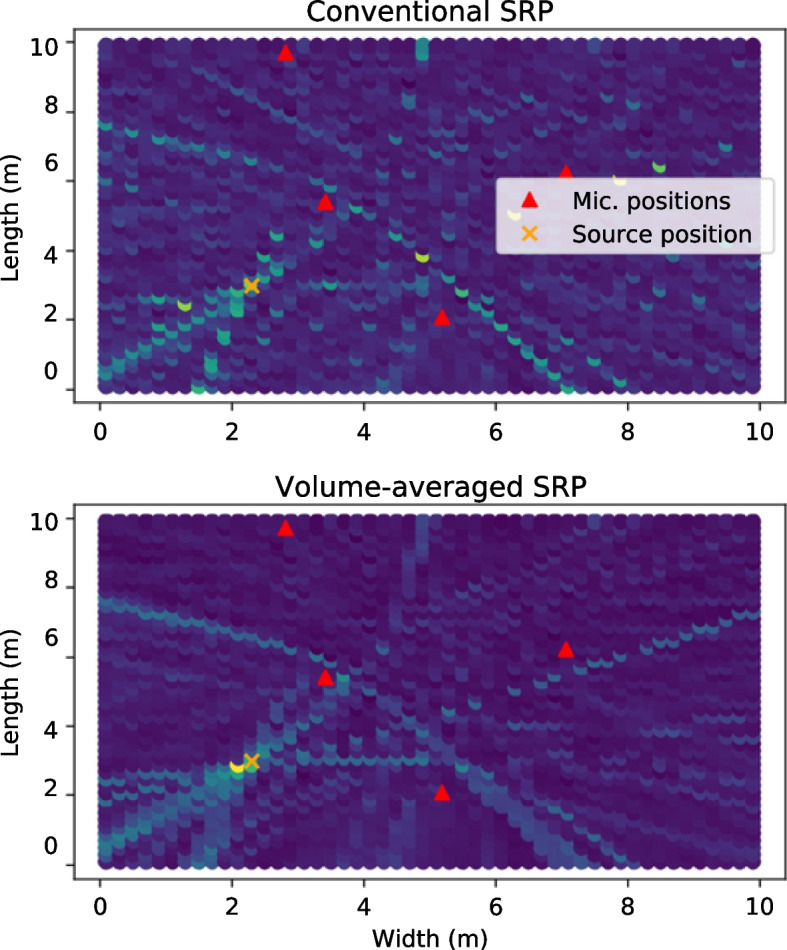
Comparison between SRP maps generated with (bottom) and without (top) volumetric techniques

As grids become coarser, each point is associated with an increasingly larger spatial region or volume. It is therefore reasonable to devise a way to modify SRP’s operation to take into account the entire set of points around the candidate. We shall refer to this strategy as Volumetric-SRP (V-SRP) methods. An example comparison between conventional and volumetric SRP maps is shown in Fig. 4. The volume surrounding a candidate position is defined as

23 $$\begin{aligned} \mathcal {V}(\textbf{u}) = & \{ \nonumber \\ & [x \; y \; z]^T \; \vert \; \nonumber \\ & | x - u^{(1)} | \le r^{(1)}/2 \nonumber \\ & | y - u^{(2)} | \le r^{(2)}/2 \nonumber \\ & | z - u^{(3)} | \le r^{(3)}/2 \nonumber \\ \}, \end{aligned}$$

where $r^{(1)}$, $r^{(2)}$ and $r^{(3)}$ respectively represent the width, length and height of the volume. The Volumetric-SRP (V-SRP) approach is typically defined by considering the SRP value of all points within the volume, which are then combined using a pooling function such as summation. The pairwise V-SRP function can thus be defined as

24 $$\begin{aligned} \text {V-SRP}_{lm}(\mathcal {V}; \mathcal {X}) = \sum \limits _{\tau \in \mathcal {V}_\tau } \textbf{g}[\tau ; \textbf{x}_l, \textbf{x}_m], \end{aligned}$$

where $\mathcal {V}_\tau$ is the set of TDOAs associated to volume $\mathcal {V}$. Different approaches and approximations can be used to selecting $\mathcal {V}_\tau$. The first approach to propose the usage of coarse grids and (24) is the Modified SRP (M-SRP) method [40]. In [40], the elements of $\mathcal {V}_\tau$ are defined by first remarking that the minimum and maximum TDOA limits in the volume must be contained in the volume’s boundary due to the hyperboloidal nature of TDOAs. These values are then approximated using the TDOA’s gradient vector and the centre of the volume. $\mathcal {V}_\tau$ is then defined as all available TDOA values between those limits. Note that when the number of TDOAs differ between candidate volumes, the algorithm’s quality may be reduced. A strategy for mitigating this is using average [41] or max [42] pooling instead of summation in (24).

The work of [43] proposes exacts bounds for the maximum and minimum and maximum TDOA limits used in the M-SRP algorithm [40] in anechoic conditions. In particular, the authors show that the minimum and maximum TDOAs of a cuboid volume can be always found by searching a set of only 26 points involving its vertices, edges and faces. Furthermore, this can be further approximated by searching only the volume’s 8 vertices, further simplifying finding the maximum and minimum TDOAs as these limits can be precomputed for any given cuboid and microphone array locations. The computational complexity of M-SRP (24) can be further reduced through an iterative subdivision of the maximal volume [41, 43–45].

### Iterative grid refinement

A common strategy used in conjunction with coarse grids consists of iteratively modifying the initial search grid $\mathcal {G}(0)$ based on the candidate position’s SRP values, allowing for the algorithm to ‘focus’ on promising regions. This procedure can be applied repeatedly until a stopping condition is reached, i.e.

25 $$\begin{aligned} \mathcal {G}(i) = \text {ITERATE}(\mathcal {G}(i - 1)), \end{aligned}$$

where the $\text {ITERATE}$ function usually involves evaluating the SRP function on the current grid candidate points, discarding points based on a criterion, and generating additional candidates based on some heuristic.

This iterative procedure may be performed using a quadtree [46, 47], a tree-based data structure commonly used for image processing. In [47], each cell of an initial azimuth-elevation square grid of size $16\times 16$ is iteratively subdivided into four non-overlapping cells, where the SRP function is computed on each region’s centre. To prevent the grid size from growing exponentially, only the cells with the highest SRP value are selected for further division.

**Fig. 5:**
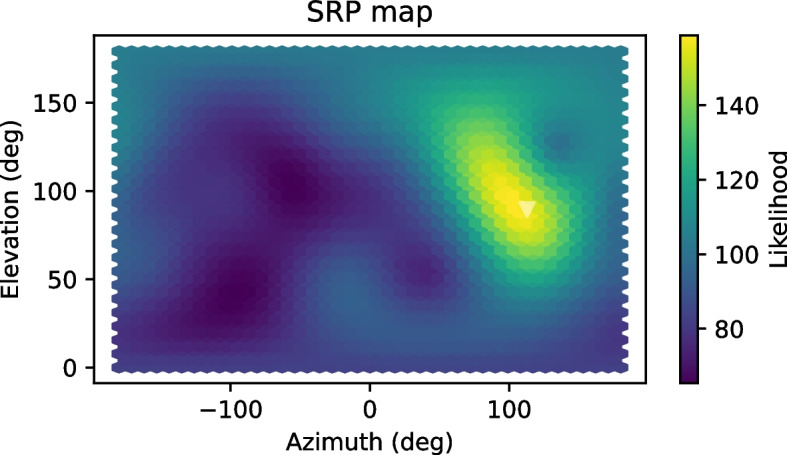
Low-pass version of the frequency-domain SRP, where only frequencies up to 200 Hz are considered

When a coarse grid is used, the true source location $\textbf{u}$ may lie on a grid point. A strategy to ensure $\textbf{u}$’s neighbours exhibit a high SRP value in initial iterations was proposed in [47], which identify that the width of a peak on an SRP map is inversely proportional to the source’s carrier frequency. Therefore, computing SRP using only low frequencies produces a smoother map. This is illustrated in Fig. 5, where only frequencies below 200 Hz are used for $\mathcal {F}$, which can be compared to Fig. 3 which shows a map generated using all frequencies up to the Nyquist rate.

**Fig. 6:**
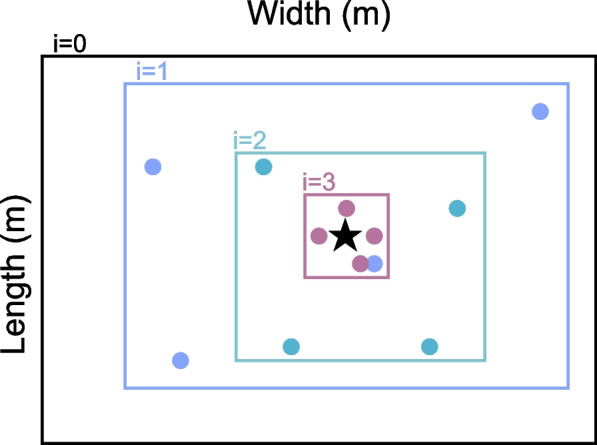
Iterative region contraction procedure, where different colours represent search regions and grids of points related to iterations i. The true source location is represented by the black star

The initial grid can also consist of points randomly sampled on the room’s boundaries, as formulated in the Stochastic Region Contraction (SRC) method defined in [48]. The region contraction procedure is exemplified in Fig. 6. The subsequent grid can be chosen by resampling a set of points on the smaller boundary containing the previous candidates exhibiting the highest SRP values. This procedure may continue for a maximum number of iterations, or until a minimum search cuboid is obtained. Note that this contraction procedure can also be applied to deterministic grids. In this case, the SRP variant is referred to as Coarse-To-Fine Region Contraction (CFRC) [49].

Although the aforementioned methods significantly accelerate the computation of SRP, they provide no guarantees that the true source location will not be discarded, as they assume the SRP map to be a concave function with its maximum at the source location. The authors of [43, 50, 51] propose a procedure which theoretically guarantees not to discard the point maximizing the SRP function in anechoic conditions using the branch-and-bound iterative search method. The search starts by considering the entire search volume, typically the entire room, and subsequently divides it into smaller volumes using a branching function. Volumes are discarded through the aid of a bounding function similar to the bounds computed in (24).

Other iterative techniques used for SRP include the Artificial Bee Colony [52], Majorization-Minimization [53, 54] and Lagrange-Galerkin [55] search methods.

### Grids based on prior location estimates

Alternatively, smaller grids can be built using lower-complexity, but less reliable source location estimators, such as those obtained using two-step methods. These candidates can then be more robustly selected and refined using SRP. In [56], the grid is initialized using the positions associated with the signals’ highest GCC-PHAT peaks, which can be interpreted as estimates of the source’s TDOA. These estimates are used to triangulate candidate source positions using a least squares approach, which are then evaluated using SRP. In [56], four peaks per pair were deemed to yield the best results.

As triangulation-based estimates are not robust to noise and reverberation, it is useful to include neighbouring points in the candidate grid, so as not to limit the performance of SRP. This can be achieved by sampling points in the cuboid region containing these candidates [57, 58]. Similar approaches are proposed by [59–62]. Grids based on prior location estimates were also explored on practical scenarios involving Wireless Acoustic Sensor Networks (WASNs) [57, 58].

### Incorporation of prior scene information

Another strategy for reducing the grid size exploits the property that spatial regions exhibit different levels of sensitivity depending on their position in relation to the microphone array [63–67]. For instance, neighbouring candidate locations may have similar or identical sets of associated theoretical TDOAs, being therefore indistinguishable using SRP [63–65]. Those can therefore be replaced by their centroid without loss in performance [63–65].

A similar concept is proposed by [66, 67], where a non-uniform, *geometrically sampled grid*, is proposed. Based on their distance, each microphone pair within the system has a discrete set of integer TDOAs, in samples, each of which defines a hyperboloid in space. Candidate locations at the intersection of multiple hyperboloids have high definition and can therefore be more reliably used for localization. Conversely, if the source is located within a low-definition region, more grid points are used to improve its localization performance.

Alternatively, information about the environment can be included as prior information to build smaller grids. For example, for specific microphone array geometries such as the T-shaped orthogonal array used by [68], the 2D azimuth/elevation grid can be decomposed into two 1D grids, which can be independently maximized, significantly reducing the number of required SRP evaluations. In [69], a method combining SRP for both DOA estimation and PSSL is proposed and tested with a large aperture, L-shaped microphone array. SRP is first used for estimating the source’s DOA with respect to the array’s branches. This direction is used to create the initial grid of candidate locations, from which the SRC variant of SRP is employed for 3D localization. A similar two-step approach is employed in [70], where distributed microphone arrays are used for DOA estimation. The intersection of these directions is then used to estimate the source location. The computational complexity of SRP can also be reduced, for linear arrays, by combining array interpolation and polynomial root solving [71]. Alternatively, if possible source locations are known, such as seat locations in a conference room, a database of possible source locations along with their respective microphone array responses can be precomputed, thereby significantly reducing the grid size [72].

Complexity can also be reduced by reducing the number of pairwise maps computed. For instance, centralized microphone arrays of symmetrical geometries such as spherical or rectangular exhibit multiple pairs of microphones with parallel directions. Computation can be reduced at a negligeable loss in performance by only using one pair for each of those directions [73]. Conversely, microphone pair selection can also be applied to distributed microphone networks, where data transmission is a secondary constraint which should be minimized. If each device contains at least two microphones, the SRP maps can be computed and transmitted independently for each device, an economic alternative to transmitting raw signals which was shown to incur only small losses in localization performance [74].

Finally, the computation of the SRP function can be avoided by only considering candidate positions with a high associated cross-correlation based on their theoretical TDOA and GCC-PHAT between microphone pairs [31, 75]. In practice, this can be achieved by creating a hash table for each microphone pair where each key-value pair represents a TDOA and its set of possible candidate positions. The keys (TDOAs) with a low associated GCC-PHAT can then be filtered out. Finally, the table is traversed, where the SRP values for the remaining sets of TDOAs associated with a candidate position are summed to create a global SRP map.

### Paralellization

When the device computing SRP supports parallel processing capabilities, such as multiple Central Processing Units (CPUs), multiple threads or one or more Graphics Processing Units (GPUs), the method can be sped up while using its original formulation, therefore guaranteeing its optimal performance. SRP is highly parallelizable, as the evaluation of the SRP function for each candidate location is independent.

A Compute Unified Device Architecture (CUDA) implementation of SRP was first proposed in [76], where the SRP function for each candidate location was computed independently on each GPU thread. In [77], a time-domain and a frequency-domain GPU implementation of SRP using CUDA were respectively compared with optimised CPU counterparts. Results show the GPU implementations resulted respectively in speed improvements of 70 and 275 times. In [78], the implementation provided by [77] is optimised by maximizing usage of the GPU’s internal memory in favour of the host’s memory, resulting in significant speed-up in comparison to [77]. In [79], an implementation of SRP is proposed for three CUDA-enabled GPU types. In [80, 81], a GPU implementation of SRP using NVIDIA’s Jetson chip, designed for low-power mobile computing, is evaluated for multiple grid resolutions. Conversely, in [80], a CUDA implementation of SRP using multiple GPUs is presented.

In [82], SRP’s computation was vectorized using Intel’s Integrated Performance Primitives (IPP) software library, reducing CPU load by a factor of two in comparison to a baseline scalar implementation. In [83], an implementation of SRP using OpenCL, an open-source parallel computing framework compatible with multiple processors including CPUs, GPUs and Field Programmable Gate Arrays (FPGAs), is presented. Experimental comparisons with device-specific implementations of SRP reveal that the proposed implementation achieves similar performance. An efficient hardware implementation of [84] is presented in [85].

### Other approaches

In [86], an SRP method based on the singular value decomposition (SVD) is proposed. Based on (17), a matrix is defined mapping all frequency-domain GCCs to all candidate locations, whereof a low-rank approximation is obtained using the SVD. This low-rank approximation allows to first project frequency-domain GCCs onto a subspace with reduced dimensions and subsequently employing a k-d tree search scheme [87], resulting in a lower computational cost at a similar localization performance to that obtained with the conventional SRP-PHAT. The performance of this method is increased in [88], where a spectral subraction procedure is applied to the correlation matrix.

It was shown in [84] that a frequency-domain SRP map can be efficiently approximated through interpolation while critically sampling the GCCs, based on Nyquist-Shannon sampling. Such approach is formulated while accounting for the physical bound over the range of possible TDOAs for a given microphone array, as well as the assumed GCC bandlimit. Simulation results indicate that the computational cost of the proposed interpolation-based approach for obtaining the approximated SRP map can be several orders of magnitude lower than the cost of computing the conventional frequency-domain SRP map, while the localization performance is maintained. In [36], this approach is extended by optimal low-rank or sparse approximations of the interpolation matrix with scalable complexity, allowing for a more favourable complexity-performance trade-off as compared to conventional frequency-domain and time-domain SRP. Results show that sparse interpolation performs better for large array apertures, while low-rank interpolation performs better at small array apertures or a large number of microphones.

## Increasing robustness

Although SRP has been shown to provide satisfactory performance in realistic scenarios [16], its performance is reduced in challenging scenarios including high reverberation and/or noise. Localization performance is often inversely related to the strategies presented in Section 3, as fine grids provide better resolution. However, other techniques are required to remove artifacts caused by noise and reverberation from the SRP maps.

### Modified GCC-PHAT functions

The quality of SRP is dependent on the quality of the cross-correlation between microphone pairs. Most approaches employ GCC-PHAT to obtain the correlation information, as it was shown to outperform temporal CC [13, 14]. Nonetheless, modifications can be employed to improve GCC-PHAT in challenging scenarios. One of such modification is $\text {GCC-PHAT}_\beta$, a parameterized version of $\text {GCC-PHAT}$ which was shown to improve localization performance, defined as [89–92]

26 $$\begin{aligned} \text {GCC-PHAT}_\beta (f ; \bar{\textbf{x}}_l, \bar{\textbf{x}}_m) = \frac{\bar{\textbf{x}}_l(f)\bar{\textbf{x}}^{*}_m(f)}{|\bar{\textbf{x}}_l(f) \bar{\textbf{x}}_m^*(f) |^\beta + \gamma }, \end{aligned}$$

where $\gamma$ provides numerical stability, and $\beta$ controls the relevance attributed to the signals’ magnitudes. Note that conventional GCC-PHAT is achieved when $\beta = 1$, whereas conventional CC is obtained using $\beta = 0$. The experiments in [90] show that intermediary values of $\beta$ (e.g., $\beta =0.8$) improve localization of narrowband signals under the interference of directional noise sources at low Signal-to-Noise Ratios (SNRs). Although $\gamma$ is often set to a small value to prevent a null denominator, Shen et al. [91] propose setting $\gamma$ to the minimum *coherence* between the signal pair over all frequency bins. Coherence is here defined as the ratio between the signals’ cross- and auto-spectral densities. In [93], the authors perform an experimental analysis of the $\text {SRP-PHAT}_\beta$ method and they verify the simulation study in [90] which shows the acceptable range of values for the partial whitening parameter $\beta$ for a general signal to be between 0.65 and 0.7. They also point out that the experiments exhibit more significant performance fluctuations for especially $\beta =1$ corresponding to the conventional PHAT method. This outcome supports the use of the partial whitening over the conventional PHAT.

An alternative to PHAT filtering consists of using the kurtosis of the signal pair, motivated by the assumption that noise is frequently modelled as a Gaussian random process, which is theoretically eliminated in the kurtosis computation [94]. The GCCs can also be replaced by a sum of Gaussians centred at the former’s most prominent peaks, thus producing a smoother SRP map [95]. The effects of the phase transform can also be replaced by a linear predictor incorporating sparsity constraints [96]. The Multichannel Cross-Correlation (MCCC) function [97] can also be employed [98]. Instead of providing a single correlation value for two signals and a delay $\tau$, MCCC provides a correlation value for a vector of *M* signals and a vector of delays $\pmb {\tau }$. The MCCCs can therefore be used to construct a beamformer which is applied as a preprocessing step before SRP [98]. Finally, the CC between microphone pair signals can be computed using an eigenvalue decomposition of the cross-correlation matrix of the microphone signals. Instead of computing the CC between microphone signals, the correlation between corresponding eigenvectors can be used, ignoring directions related to noise and reverberation [99] and therefore improving the quality of the SRP map.

The GCC-PHAT function of a broadband signal in an ideal, anechoic scenario is an impulse with its main peak occurring at the microphone pair’s TDOA. However, as the source signal becomes narrowband, the pair’s GCC-PHAT becomes a sinc function ($\text {sinc}(x) = \sin (x)/x$), i.e. a function exhibiting multiple ripples which translate into low-quality SRP maps. In this case, the envelope of the Generalized Cross-Correlation (GCC) function, obtained by extracting the magnitude of its analytic signal, can be applied instead to remove the aforementioned ripples.

In other broadband cases, some frequency bands may be more affected by noise than others. In those cases, it is advantageous to analyse the CCs in different frequency bands. This is done, for example in [100], which proposes the creation of a GCC matrix, where columns represent frequency bands and rows represent time delays. The conventional GCC-PHAT can be obtained from this matrix as long as the Constant Overlap-Add principle is satisfied when selecting the frequency band centres and widths. The authors show that degradations from noisy frequency bands can be reduced by applying SVD to obtain a low-order approximation of the GCC matrix, improving the robustness over the conventional GCC-PHAT.

Many challenges also arise when applying SRP in large outdoor environments. Firstly, these environments suffer from intense low-frequency environmental noise, often requiring the signals to filtered before processing, thus creating a band-passed input signal which introduces challenges for the SRP method as described in [101]. Secondly, the size of the search area may require very large grids, significantly increasing the method’s computational cost. Finally, factors such as changes in temperature, terrain, wind and position of the sensors make the propagation time model defined in (7) unreliable. The authors of [102] propose a modified GCC function based on Wavelet theory which takes the three aforementioned factors into account to improve the performance of SRP in outdoor environments.

Finally, the GCC-PHAT function can be substituted by a neural network [103], as will be discussed in Section 4.4.

### Improving combination

The formulation defined in (17) combines pairwise and frequency-wise SRP values through unweighted summation. A more general formulation of SRP, which we denote Weighted SRP (W-SRP) can be written as

27 $$\begin{aligned} \text {W-SRP}(\textbf{u} \, ; \,\, \bar{\textbf{X}}) = \bigcup _{(l, m) \in {M \atopwithdelims ()2}} \bigcap _{f \in \mathcal {F}} \frac{\text {SRP}_{lm}(\textbf{u}, f \, ; \,\, \bar{\textbf{x}}_l, \bar{\textbf{x}}_m)}{k_f k_{lm}}, \end{aligned}$$

where $\bigcap$ represents the operation combining frequency information, $\bigcup$ represents the combination of pairwise information, and weighting factors $k_f$ and $k_{lm}$ respectively weight frequency and pairwise information. Besides classical summation, choices for the pairwise combinator $\bigcap$ are the product $\prod$ and the Hamacher t-norm, among others [104]. Conventional SRP combines pairs through summation, meaning that pairwise SRP maps combined in such manner will exhibit high values if any pair does so. Conversely, if multiplication is used, all pairwise maps must exhibit high values for the global SRP to do so. In an extreme case, if any pairwise map is null, so will be the global SRP map. The simulated experiments in [104] show that combining pairwise SRPs through their product results in a significant increase in localisation performance over their sum, reducing the localization Root Mean Squares (RMSs) error by $45\%$.

The weights $k_{lm}$ can be computed on pairwise SRP maps, for example, from a fractal theory standpoint, giving less importance to noisier, pairwise SRPs [105], or by measuring the noise of the GCC-PHAT vector by computing the ratio between the GCC-PHAT’s peak and its average [106]. Note that microphone pair selection is also included in (27) for the special case $k_{lm} = \infty$.

Conversely, the frequency weight $k_f$ can be set as the maximum SRP value across all pairs, therefore equalizing the contribution of each frequency bin to the global SRP. This is shown to offer a similar effect to the PHAT weighting [107]. Another approach estimates $k_f$ using neural networks [108–111], as will be discussed in Section 4.4.

### Pre/post-processing

Applying pre- or post-processing to the microphone signals in search of anomalies may improve SRP maps. For example, a Voice Activity Detector (VAD) can be used to detect the presence of speech in a noisy environment, in order to prevent SRP from unintentionally localizing noise sources [112], or to improve the localization of impulsive sources [113]. A VAD can also be used to discard directional noise sources [114]. SRP maps can also be improved through the application of a Wiener filter [106].

### Neural approaches

As in many other tasks in acoustic signal processing, neural networks have also been applied for the task of SSL, frequently obtaining state-of-the-art results in comparison to classical methods such as SRP [15]. However, SRP still presents several advantages over classical neural network methods, which usually require matched training/testing microphone geometries. Furthermore, SRP maps serve as an excellent input feature for neural networks. Finally, SRP’s building blocks can be advantageously replaced by neural blocks, bridging the gap with neural methods’ performance in challenging environments. The approaches below are related to the strategies mentioned in the above subsections.

One of such blocks which can be improved is GCC-PHAT. A deep neural block can be used to estimate an idealized GCC-PHAT vector which removes peaks associated with reverberation and noise. A Deep-GCC function can be formulated in the time [103, 115] or frequency [116] domain. In the time domain, the Deep-GCC vector should exhibit a single peak at the source’s true TDOA $\tau _{lm}$, modelled as a Gaussian with standard deviation $\sigma _d$ as [103, 115]

28 $$\begin{aligned} \text {Deep-GCC}(\tau ) = \textrm{exp} \left( \frac{-|\tau - \tau _{lm}|^2}{2\sigma _d^2} \right) . \end{aligned}$$

In practice, (28) serves as the target loss function for the network being trained. The choice of input feature and architecture for a Deep-GCC function may vary. In [103, 115], GCC-PHAT itself is chosen as the networks’s input and a 1-D Convolutional autoencoder is selected as architecture. In [116], the magnitude and phase spectrograms of both microphone signals are chosen as the input features, and a Convolutional Recurrent Neural Network (CRNN) is chosen as the neural architecture.

Many approaches focus on using neural networks to estimate a weighting function, similarly to the signal processing based procedures described in Section 4.2. Most approaches focus on the frequency weights $k_f$, inspired by the task of speech enhancement, where neural time-frequency masks have attained significant success [108]. For instance, a Convolutional Neural Network (CNN) can be trained to estimate a time-frequency mask to reduce the interference of directional sources, using the output of a Wiener filter as its target function [108]. Other targets can be used, such as the distance between the true and SRP-estimated locations for a single frequency band [109, 111]. Alternatively, the SNR on each microphone can be used as a weight for each frequency band [110]. Similar approaches been also employed other machine learning methods, namely, Support Vector Machines (SVMs) and Radial Basis Function Networks (RBFNs) [117–119].

Another prominent manner of improving localization performance using SRP uses the SRP maps as the input feature of a deep neural network. In this case, the neural network may have two goals: to enhance the maps produced by SRP [120, 121], and/or to extract the source locations using the map [17, 85, 122–125], i.e. to improve the grid search/peak-picking function defined in (20). The networks differ in the architecture used, such as the Multi-layer Perceptron (MLP) [122, 125], 3D [17, 85, 123], spherical [124] and icosahedral [120, 121] convolutions.

**Fig. 7:**
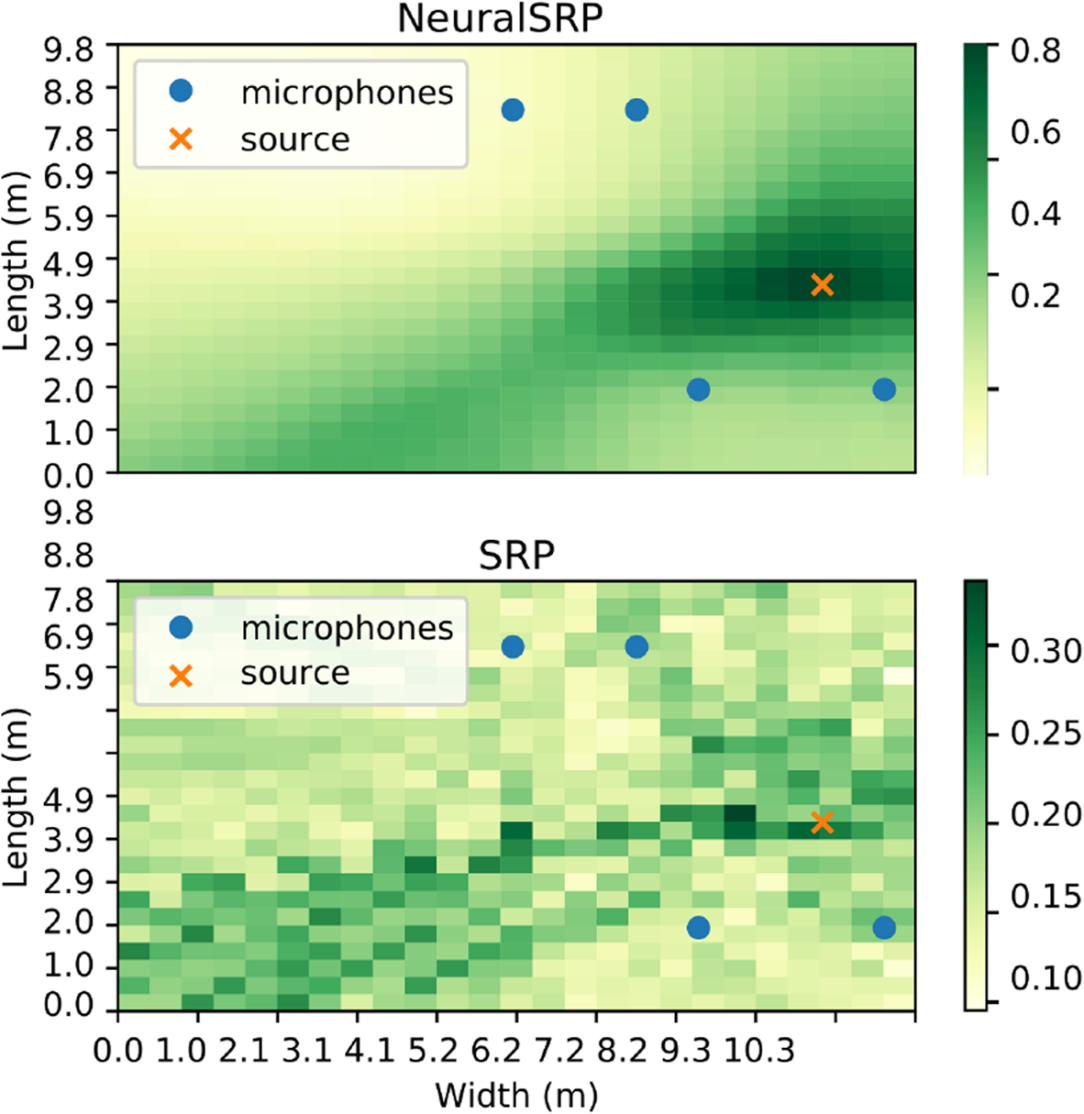
Neural-SRP+ [126] and conventional SRP map in a highly reverberant room. The source position is shown with a cross and the microphone positions with circles

Finally, other neural approaches simulate the pairwise processing used by SRP for the task of source localization. The authors of [125] remark that the SRP algorithm shares architectural similarities with the Relation Network, a type of Graph Neural Network (GNN). In the context of SRP, a *relation* between two microphones consists of the pairwise SRP maps shared between them. All pairwise relations are then summed, creating a global relationship between all microphones, which can be used to estimate the source locations. Neural-SRP approaches [126, 127] therefore replace SRP’s function with a neural network, reducing the detrimental effects of noise and reverberation by including challenging scenarios during network training. An example of a map produced using a Neural-SRP method is shown in Fig. 7.

### Other approaches

SRP maps can also be analysed by decomposing them using a set of idealized pairwise maps, computed using the theoretical TDOA between the microphone pairs and the candidate locations. Instead of estimating the source location through peak-picking, the search can be done by matching the pairwise SRP maps with a subset of idealized maps according to a similarity metric [26, 128].

When the distance between microphones in a centralized array is small, so is the range of possible TDOAs between the pair as expressed in (10). It is therefore desirable to perform interpolation in the CC function to obtain sub-sample TDOA resolution when using the temporal SRP formulation. The work of [35] evaluates the performance of SRP for DOA estimation from concert hall recordings using three different interpolation methods, namely, parabolic, exponential and Fourier. The study reports best performance using exponential peak interpolation.

The work in [129] presents a system where, before performing localization using SRP, a speaker verification step to remove unwanted speakers and noise is applied.

In [130], the authors exploit spatial diversity in order to improve SRP’s performance in reverberant environments. Their simulation results show that large arrays are affected by the reverberation more than smaller ones and that having a smaller distance between microphone arrays results in more accurate localization. When the number of microphones in an array is increased the localization results are more robust as expected, but separating it into two array makes it even more favourable compared to merely increasing the number of microphones in a single array.

In [131], a mel-frequency extraction technique is employed with SRP-PHAT in order to obtain an enhancement of human speech and process it more robustly in a noisy environment. As a performance metric, peak SNR (PSNR) is used. The results show that utilizing Mel-frequency Cepstral Coefficientss (MFCCs) in conjunction with SRP-PHAT yields higher PSNR values compared to using only the SRP-PHAT, which results in a more accurate localization.

In [132], the authors compare the SRP-PHAT localization performances using a Uniform Linear Array (ULA) and a Coprime Microphone Array (CPMA) interleaving two linear arrays with coprime dimensions. They show that a CPMA offers better localization results than a ULA with the same number of microphones. In another study [133] by the same authors, a performance analysis of Semi-Coprime Microphone Arrays (SCPMAs) for localization using the SRP-PHAT algorithm is conducted. They evaluate the performance in terms of beam pattern, array gain and DOA estimation. The results on beam pattern an array gain suggest that the SCPMA outperforms the CPMA in reducing the peak side lobe level and minimizing the total side lobe area. Moreover, it shows an enhanced ability to amplify the target signal while suppressing the noise. The results of DOA estimation in anechoic and low reverberant environments show that the SCPMA delivers accurate estimates which are on par with the estimates obtained from the full ULA. However, in highly reverberant conditions such as a 400 ms reverberation time, side lobes in the beam pattern of the SCPMA result in less accurate estimates.

As discussed in Section 2.1.1, the range $\rho$ can only be accurately estimated when the source is located in the near-field with respect to the microphone array. The field type can be estimated by comparing the SRP of two circular candidate grids at different distances, one in the far-field, the other in the near-field. The grid exhibiting the highest SRP value dictates the field regime. If near-field conditions are found, a second SRP grid search can be applied for range estimation [134].

## Multi-source SRP approaches

We start this section by revisiting the problem statement described in Section 2. Instead of defining the target output of our system as a single source position vector $\textbf{u}$, we extend it to be a matrix $\textbf{U}$ of dimensions $3 \times N$, defined as

29 $$\begin{aligned} \textbf{U} = \left[ \begin{array}{llll} \textbf{u}_1&\textbf{u}_2&...&\textbf{u}_N \end{array}\right] , \end{aligned}$$

where *N* is the number of active sources. Note that *N* is usually unknown in practice and must also be estimated on such cases. Updating the model for the signal received at each microphone is also required, as it becomes a weighted sum of all active sources. In the frequency domain, the received signal at microphone *m* can be described as

30 $$\begin{aligned} \bar{x}_m(t, f) = \sum \limits _{n=1}^N s_n(t, f)a_{m}(\textbf{u}_n, f)e^{-jf\tau _{m}(\textbf{u}_n)} + \epsilon _m(t, f). \end{aligned}$$

Despite the modified signal model, the analysis of the CC function between two microphone signal frames $\textbf{x}_l$ and $\textbf{x}_m$ in the presence of *N* simultaneous talkers usually presents one peak related to each source. Although this would allow the conventional SRP method to be used directly, the function may also exhibit ‘ghost peaks’ related to the reflections caused by the room’s surfaces, hindering the estimation procedure. Also, the relative amplitude of peaks may vary considerably, especially in cases where the sources have different power levels, hindering the application of simple thresholding methods. Finally, the interfering sources reduce the correlation amplitudes at delays $\tau _{m}(\textbf{u}_n)$ are reduced in comparison to the single source case, hindering the analysis of the SRP map.

Due to the aforementioned limitations of using the conventional SRP method for multi-source localization, different SRP-based alternatives have been proposed. These alternative methods, while presenting their own particularities in terms of implementation, target scenario and performance, are categorized in the following subsections based on their core modification when compared to the conventional SRP method.

### Modified SRP computation

As straightforward alternatives to the use of conventional SRP for localizing multiple sources, different strategies proposed in the literature focus on simply modifying the process of computing the SRP map. For instance, in [90], a parametric modification of the PHAT-weighting function is proposed, aiming to achieve flexibility in combining different narrowband components. Simulation results, obtained for both single and multi-source cases, indicate that the use of the modified PHAT-weighting function can improve localization performance for both narrowband and broadband signals.

In [135], similarly to the efforts aimed at achieving an improved combination of pair-wise information for increasing localization robustness outlined in Section 4.2, the use of harmonic and geometric means of the GCC functions over all available microphone pairs was explored to build an acoustic map. When compared to the conventional summation of pair-wise functions, as previously expressed in (15), results show that the use of geometric and harmonic means contributes to removing undesired sidelobes and improving source level estimation.

### Source cancellation

**Fig. 8:**
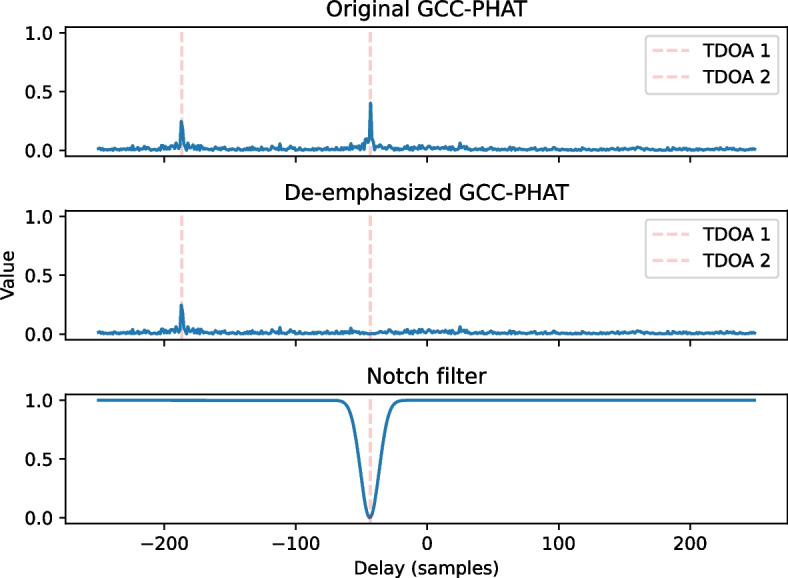
Representation of the de-emphasis procedure described by Brutti et al. [28]

Another class of alternative methods for multi-source localization aim to exploit the observed robustness of SRP in single-source scenarios by relying on schemes that reduce the influence of a previously located and dominant source on newly computed SRP maps, which will be here referred to as the process of source cancellation. For instance, in [28], the localization of two sources is performed in a two-step manner. First, the position of the source with the highest correlation peak is estimated as in the conventional SRP method. To estimate the second source, the first source is de-emphasized from the CC function through the use of a TDOA-domain notch filter. This process is illustrated in Fig. 8. Although this approach can be further applied for the localization of three sources, the authors state that the noise in the correlation function with three sources would be prohibitive, and that tracking approaches should be applied instead.

The removal of a previously located source’s contribution from an SRP map can also be achieved through the projection of the observed GCCs onto a subspace that is orthogonal to the source position, as described in [136]. Results obtained with both simulated and experimental data indicate that such an approach can outperform the de-emphasis method from [28], especially in cases of sources with different power levels. Moreover, the use of a truncated formulation of the proposed source cancellation scheme allows for a reduction in computational cost while performing comparably to [28], without requiring parameter tuning associated to the TDOA-domain notch filter design.

Subspace processing for source cancellation within an SRP-based framework has also been proposed in [137], where the SVD-PHAT method [86] is extended to address the case of multiple sources. Thererin, the contribution of a previously located source (obtained by means of a k-d tree search) is removed from the observed projections of the GCCs onto a reduced-dimensional subspace. The proposed multi-source SVD-PHAT approach was compared to a source cancellation scheme, similar to the de-emphasis method from [28], where a source’s contribution is removed from the observed GCCs and a new SRP-PHAT map is computed for locating the next source. Simulation results indicate that the multi-source SVD-PHAT can outperform the successive recomputation of the SRP-PHAT map.

As an alternative to employing a source cancellation procedure to the observed GCCs, the spatial gradient SRP-PHAT method proposed in [138] involves successively removing the influence of the current, most dominant source directly in the observed SRP map by means of a negative spatial gradient function. Experimental results for two-speaker scenarios show that the spatial gradient SRP-PHAT can be an effective localization method in scenarios with a diffuse noise field.

In [139], an approximate analytical formulation of an SRP map using a Gaussian Mixture Model is proposed, such that probability density functions can be used to estimate the location of multiple sources while removing their corresponding contributions from the probabilistic SRP map. Experimental results with scenarios involving up to three speakers indicate that while this approach can effectively locate multiple sources, its performance degrades when sources differ greatly in power.

Based on the multi-source localization alternatives briefly discussed above, we note that, overall, combining SRP with source cancellation schemes demonstrates potential in extending the robustness of SRP from single-source to multi-source scenarios. However, we also note that these approaches rely on iterative procedures, which might not be suitable for all applications.

### Grid refinement

In addition to source cancellation, grid refinement schemes have also been combined with SRP for multi-source localization. This concept is generally motivated by the possibility to balance the advantages of evaluating the SRP function over high-resolution grids of candidate positions and the overall computational cost. As an extension of [139], grid refinement is indirectly used in [140], where different zones of interest, defined in terms of TDOA intervals, are identified as those where acoustic sources are dominant, based on a cumulative SRP function. Thus, a conventional grid search step for source localization can be performed over a reduced search space with the desired spatial resolution. The localization of multiple sources can then be achieved by iteratively removing the influence of the dominant sources via the probabilistic scheme from [139]. Experimental results show that such approach can improve localization performance in multi-source scenarios at a lower computational cost than the authors’ previous work.

Alternatively, in [141], a hierarchical search-grid refinement method is proposed, where a probability measure of a sound source’s presence in different regions, formulated as a spatially averaged SRP map, is used to identify the limited set of steering directions for which the search grid resolution can then be improved for localizing multiple sources. This approach is shown to lower the computational cost while performing similarly to the conventional SRP method that employs the highest resolution level over the entire search space.

### Clustering and distance analysis

Another concept often exploited in multi-source localization methods relates to data clustering and analysing distances between multiple source location estimates. For instance, the sources’ preliminary location estimates can be obtained through the conventional SRP method. Then, spatial clustering can be employed to track the estimated locations of multiple sources over different time frames [142]. Alternatively, a narrowband SRP formulation can be employed to obtain location estimates per frequency bin and time frame, while Gaussian mixture modelling can then be used to cluster the location estimates [143]. Furthermore, both the location and activity of multiple sources can be tracked [143].

In [144], source location estimates are obtained by using SRP-PHAT combined with agglomerative spatial clustering and SRC (cf. Section 3.3). Experimental results show that the localization performance of the proposed approach degrades when the peaks to be identified have widely different amplitudes or are closely located in the CC function. Accordingly, the proposed approach is further extended in [145], by replacing the agglomerative clustering step from [144] with Gaussian mixture modelling of the observed SRP map, or by identifying the peaks in the SRP map while assuming a minimal distance between sources. The performance limitations first demonstrated in [144] are also addressed in [146], where the localization of multiple speech sources is achieved by computing subband SRP maps, estimating the dominant source’s position for each subband, and employing agglomerative clustering across all subbands to obtain the final set of source location estimates. In [147], a method named Multi-Stage Rejection Sampling (MSRS) is proposed, which involves spatially clustering probability density points, derived as a function of the observed SRP-PHAT map, to identify regions of interest. Then, volume contraction is used in the identified regions for localizing multiple sources.

In [148], a three-step framework is proposed for multiple source localization. It relies on: step 1) partitioning the search region into cubic volumes, clustering such volumes and, based on equivalent TDOA bounds; step 2) computing a delay density map to find in which clusters it is more likely to have a sound source; step 3) further analysing the chosen clusters with conventional SRP to obtain the final source location estimates.

Finally, the approach proposed in [149] for a specific microphone setup of central and lateral microphone arrays, involves finding the intersection between the source positions estimated with the central array’s SRP map and the ones estimated with the lateral arrays through an adaptive subband generalized eigenvalue decomposition (GEVD) scheme, in order to obtain the final 3D location estimates of multiple sources. Simulation results with up to three speakers demonstrate that the proposed method outperforms other state-of-the-art methods under varying levels of noise and reverberation.

### Sparsity assumptions

In a wide range of applications, the task of source localization is set to be performed in scenarios that allow for the assumption of a limited number of active sources with respect to the search grid of candidate locations being employed. This has motivated sparsity-based modifications to the conventional SRP method to also be proposed, given the potential to represent observed acoustic maps with more distinct peaks, and consequently, improve multi-source localization performance. For instance, localization can be performed by employing a sparse-regularized generative model that fits the observed SRP map, combined with a subspace filtering step that compensates for what is not directly accounted for by the fitted model [128]. Experimental results show that although the use of this approach can outperform the conventional SRP-PHAT in the multi-source scenarios tested, its overall performance highly depends on the choice of the hyperparameters used in the proposed problem formulation.

Alternatively, in [150], it was shown that group sparsity can be exploited when modelling an observed broadband SRP map as a linear function of power spectral densities (PSDs), related to an overcomplete set of candidate locations. Hence, multi-source localization can be achieved by solving a group-sparse optimization problem and identifying peaks in the estimated PSDs. Simulation results obtained for two-speaker scenarios showed that the proposed method performs better than or similar to the conventional SRP-PHAT method for varying levels of noise and reverberation, while overall outperforming the frequency-domain Sparse Iterative Covariance-based Estimation (SPICE) [151, 152] method. The method proposed in [150] was further extended in [33], by allowing for the resolution of the search grid used to compute the observed SRP map to be lower than the one defining the PSD vector to be estimated and used to localize multiple sources. Additionally, a reformulation of the group-sparse optimization problem from [150] was proposed to facilitate its regularization tuning [33]. Results obtained with simulated and experimental data showed that the extended method presented an advantage in locating closely spaced sources when compared to the SRP-PHAT and other state-of-the-art methods, especially in reverberant scenarios. Furthermore, potential approaches to reduce the method’s overall computational complexity while maintaining its localization performance were also explored.

As opposed to the methods presented in [33, 128, 150], in which spatial sparsity of acoustic sources is assumed, the method proposed in [153] demonstrates the possibility to exploit time-frequency sparsity for localizing multiple sources. Therein, it is assumed only one speech source is dominant in a given time-frequency bin, i.e. they are assumed to be W-disjoint [154]. By analysing each frequency bin and performing single-source localization, histograms with all individual DOA estimates can be generated and used in a matching-pursuit-based step of the proposed localization scheme. Simulation and experimental results indicate that this approach can outperform other state-of-the-art multi-source localization methods, at a lower computational cost. The sparsity of speech signals in the time-frequency domain is similarly exploited in [155], where a weighted, wideband histogram of source locations is computed based on narrowband DOA estimates, obtained with SRP-PHAT applied to different frequencies and observation frames. The weighted histogram is then used to perform multiple source localization through peak detection, and simulation results indicate the advantage of the proposed method when compared to the wideband SRP-PHAT for two-speaker scenarios in reverberant environments.

In [156], it is shown that sparse modelling of the GCCs observed by a microphone array can also be employed in the task of localizing sound sources and their corresponding acoustic reflections. A linear inverse problem is proposed to be solved, with its formulation depending on a time-domain propagation matrix. The authors present two implementations of the proposed method, with the first based on orthogonal match pursuit (OMP) [157], and the second on the truncated Newton interior-point method [158]. It is demonstrated through an experimental study that the use of sparsity constraints in the solution of the proposed linear inverse problem contributes to better location estimates when compared to the direct use of a time-domain SRP map. The choice of propagation matrix used for formulating the linear inverse problem presented in [156] was further investigated in [159], where the influence of the temporal width threshold, associated to the determination of propagation matrix coefficients, is demonstrated. Additionally, when assuming the GCC coefficients to be PHAT-weighted, an alternative formulation of the propagation matrix circumventing such temporal width threshold is proposed, with experimental results indicating the advantage of using such alternative formulation in terms of computational time.

Finally, in [160], an SRP-based method is proposed for simultaneous multiple source localization that employs Non-negative Matrix Factorization (NMF) [161] to decompose the time-frequency signal into a weighted sum of broadband atoms, which are time-frame-dependent and correspond to different groupings of frequency bands related to distinct sources. This method, named SRP-NMF, attempts to combine the advantages of both narrowband and broadband approaches that exploit sparsity in their corresponding domains, and experimental results indicate it performs better than or similarly to state-of-the-art methods based on fully broadband or narrowband signal formulations.

As presented above, sparsity-based modifications to the SRP method have been extensively explored and shown to improve localization performance in multi-source scenarios. However, it should be noted that the observed benefits confirming their practical relevance generally come with increased computational complexity in comparison to the conventional SRP framework. Therefore, this not only highlights the need for analysing available resources when selecting a multi-source localization method to be deployed, but also reaffirms the relevance of SRP as a foundational method for further developments targeting multi-source scenarios.

## Practical considerations

### Applications

SSL is a foundational task which has been applied in many domains, having been used as an input feature for speech enhancement/beamforming tasks [162–164], voice activity detection [165, 166], speaker diarization [167–171], sound source separation [6, 7, 172] and array calibration [173]. Furthermore, SRP’s localization performance can be improved by combining it with other sensors, such as LIDAR [174, 175] or multi-sensor devices [176]. SRP has been used on the multiple practical scenarios described below.

Although SRP can be used to localize any type of sound source, many applications focus on a specific sound event. A prominent application is that of surveillance and defence. SRP can be used to localize irregular Unmanned Aerial Vehicles (UAVs) activity [177, 178], as well as using an UAV with an embedded microphone array to localize sources of interest itself [179, 180]. Other applications in security include intrusion detection [181, 182] and gunshot localization [3, 183].

Another category of interest is that of scene understanding in large and/or outdoor environments, such as the detection of indoor and outdoor sources of noise pollution [184–186] and the detection of underground seismic events [187]. SRP was also applied for commercial and environmental purposes, such as the localization of sound-emitting fish using an underwater hydrophone [188], and to detect faulty equipment within electrical power stations [189]. Furthermore, with the increased interest in smart and self-driving vehicles sensors, localization of horns and crashes using SRP [48, 190] can also be performed, or localizing talkers inside the vehicle itself [191].

Turning to indoor environments, SRP can be applied to the medical domain, being used to localize and analyse footsteps with the goal of early detection of dementia [192], as well as for fall detection of elderly people [2]. SRP can also be used to improve human-robot interactions [193–195], as well as for camera steering corporate meetings [4] and smart rooms [196, 197]. SRP was also applied to a helmet-mounted microphone array [198], which can be used for increasing acoustic awareness on industrial sites, for example.

### Tracking moving sources

Although a source may remain mostly stationary in many scenarios such as conference calls, the same cannot be said for many situations in surveillance, robotics and healthcare. It is therefore reasonable to reformulate the source position $\textbf{u}$ to be time-dependent, i.e. $\textbf{u}(t)$. The task of estimating a source’s position at multiple time instants is hereafter referred to as tracking.

A straightforward way to achieve tracking using conventional SRP is to compute an SRP map and estimate the source position independently for successive frames at times $t_{i - 1}$ and $t_{i}$. This estimate can be often improved through the incorporation of a state-space dynamic model as well as previous estimates $\{ \hat{\textbf{u}}(t_{i - 1}) \;\; \hat{\textbf{u}}(t_{i - 2}) \ldots \}$. Such a state-space model provides source tracking by introducing dynamic constraints into the source localization procedure, modelling for instance the speed of the source. This allows for smoother position estimates to be produced and for unreliable observations, such as those caused by reverberation and noise, to be properly identified and handled.

The most common approaches for source tracking using SRP are Kalman filters [142, 199–201], particle filters [200, 202–208] and deep neural networks [17, 116, 120, 124]. Unlike in neural methods, the state-space model is explicitly defined in Kalman and particle filters.

Particle filters are frequently preferred over Kalman filters due to their simpler formulation and ability to model non-linear systems. Particle filters model the source location with the help of *Q* candidate positions known as particles, each having an associated likelihood or weight $\pi _q$, $q=1,\,... ,\,Q$. The estimated source location is obtained as a weighted sum of the particles, where the weights are their respective likelihood. At each iteration, the particles are updated according to a given kinematic model. Optionally, a resampling process may be also applied to reduce the variance of the particles.

The movement of a source at consecutive time steps is commonly modelled using Langevin dynamics [202, 204–207, 209], which assume that the source moves independently in each direction.

### Directional sources and microphones

The SRP signal model can be modified for the case where sources and/or microphones exhibit directional acoustic behaviour, that is, the amplitudes of the microphone signals are dependent on the orientation of microphones and sources. The directivity profile for microphone *m* is defined as a function $0 < d^{(1)}_m(\theta _m) \le 1$, where $\theta _m$ is an angle. An analogous function can also be defined for the source’s directivity $d^{(2)}(\theta _s)$. Finally, we define angles $\theta _1$, $\theta _2$, $\theta _3$ and $\theta _4$ as the angles of departure, the source direction, the angle of arrival and microphone direction respectively. The attenuation term defined in (2) can then be specified as [210]

31 $$\begin{aligned} a_m = d^{(1)}_m(\theta _1 - \theta _2) d^{(2)}(\theta _3 - \theta _4)\frac{k_d}{\Vert \textbf{u} - \textbf{v}_m \Vert }, \end{aligned}$$

where $\frac{k_d}{\Vert \textbf{u} - \textbf{v}_m \Vert }$ represents the attenuation caused by propagation, which generally follows an inverse law. In practice, this attenuation can be incorporated into SRP by including the source’s candidate orientation as another search dimension [210]. Note that the gains between microphones must be assumed to be calibrated, and that the source and microphone directivity patterns, as well as the microphone orientations, must be known or assumed. Microphone directivity can also be exploited to reduce the number of microphone pairs and region size used for SRP [73, 201].

When operating with distributed microphone arrays, source directivity can be estimated in two steps, firstly by estimating the source position, followed by the creation of a spherical grid around the source. The point with the highest SRP value on the sphere is selected to represent the source’s orientation [24, 25]. A similar approach is applied in [211–213], which assumes that the arrays directly facing the speaker will exhibit an SRP map with a sharp peak. The sharpness is measured using the map’s kurtosis, which is then used to estimate the talkers orientation. If the microphone gains are calibrated, the GCC-PHAT’s peak values can be used for comparison instead of the kurtosis [214].

### Comparing SRP to other approaches

In [215], the authors compare SRP to alternative real-time source localization algorithms under noisy and reverberant conditions. The alternative methods included a two-step approach and a local beamforming-based approach. The algorithms were tested under signal-to-reverberation ranges of [$-2$ dB, $-12$ dB] and SNRs of [$-6$ dB, $-16$ dB]. The accuracy of SRP-PHAT was shown to be at least 20% superior than the baselines algorithm but has a larger computational cost.

In [216], a comparison between the root-MUSIC algorithm [217], and near-field and far-field versions of SRP-PHAT algorithm is made in terms of robustness and computational load. Their results show that far-field version of SRP-PHAT has $3-15$ times higher computational load than root-MUSIC and the near-field version has $20-100$ times higher computational load. Even though root-MUSIC is more computationally efficient, SRP-PHAT was shown to exhibit superior performance in challenging conditions, such as environments with reverberation and low SNR.

In [218, 219], the authors evaluate the performance of broadband spatio-spectral estimators, including SRP and two-step localization methods. They perform an eigenanalysis of the parameterized spatial correlation matrix and show that the attenuation can be estimated from this matrix. They propose a DOA estimator based on MCCC and show that this method yields a higher resolution than the conventional SRP under low-reverberation scenarios, but worsens as reverberation increases to 600 ms.

In [220], the authors evaluate the performance of a multiple source localization method based on Augmented Intensity Vectors (AIV) using spherical microphone arrays. Their simulations showed an improved accuracy between 5 and 10 degrees for the AIV approach for sources with angular separation larger than 30 degrees. Their experiments also showed that the performance of SRP was degraded when localizing three or more sources separated by less than 45 degrees.

In [221], the authors compared two SRP variants [47, 56] previously explained in Section 3 in terms of localization performance and computational complexity. They conducted experiments in both simulated environments and real-world scenarios in which speakers were recorded by eight microphones spread out on the wall and the ceiling. Their results indicate that Hybrid Localization is more robust and computationally efficient than Hierarchical Localization in near-field, reverberant scenarios.

### Analyses of SRP

In [16], the authors show SRP to be a special case of the Maximum Likelihood Sound Source Localization (ML-SSL) method under low noise environments. The ML-SSL method described in [16] jointly models reverberation and noise as an addited Gaussian signal to the target speech. From this formulation, a Maximum Likelihood criterion is formulated, from which a map similar to SRP can be constructed. The authors also show that the Phase Transform (PHAT) is not just a heuristic but an optimal solution with a theoretical foundation under low-noise conditions.

The authors of [222] propose an analytical model based on sound propagation and its interaction with the environment that predicts SRP maps in both anechoic and non-anechoic conditions, and under both far- and near-field assumptions. They investigate how and to what extent the signal bandwidth, array topology, room geometry and spectral content of the signal affect SRP maps. The findings show that SRP functions depend on the array topology, room geometry and signal bandwidth but not on the spectral content of the signal. They validate their model by comparing it with the true SRP maps.

In [223], the authors investigated the geometrical sensor calibration errors in a ULA used in far-field human speech source localization. They observed that the errors in configuration of the endpoint sensors result in larger localization errors compared with same configuration errors of the inner sensors. In addition, they show that the localization errors increase when the total configuration error is above a threshold related to the propagation distance and the system’s sampling rate.

In [224], the authors proposed an SRP constraint to suppress local extrema. They weighted the SRP function using a coherence factor, determined by observing the signs of the GCCs between all possible microphone pairs. If the sign was the same for all microphone pairs, this indicated a high coherence and the coherence factor is 1. If half of them were negative and half were positive, then were deemed as incoherent, and assigned a coherence factor of 0. The method was shown to operate without loss of localization accuracy with respect to conventional SRP.

## X-SRP

In this section, we describe the SRP method from an algorithmic perspective, with the goal of unifying the previously described extensions of the method within a common framework. The main functionality described in Section 2 is revisited by substituting specific functions with generic ones, which we shall refer to as *modules*. For example, the CC function used in the classical SRP is substituted by a module called compute_signal_features, which can be instantiated as the temporal CC, GCC-PHAT, or a neural-based feature as in [108, 116].

**Fig. 9:**
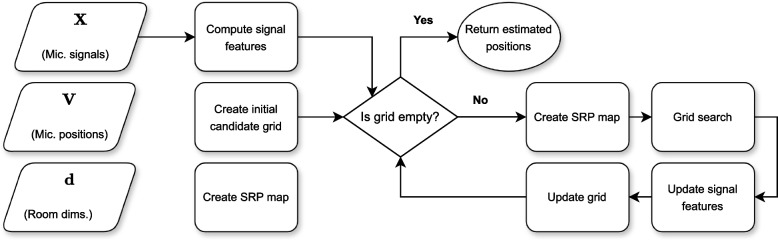
Flowchart of the generalized SRP algorithm. Parallelograms represent input data, rectangles represent functions, diamonds represent decisions and ellipses represent terminal states

This modular perspective allows for SRP papers to be grouped in an alternative way to the task-oriented manner used in the previous sections. Conversely, the categorization presented here groups the works by their implementation details, facilitating their combination and comparison.

To facilitate the reproduction of SRP variants and explore novel variants, we release the eXtensible-SRP, or X-SRP Python library, which provides a modular implementation of SRP following Algorithm 1, which is also shown as a flow diagram in Fig. 9. We include multiple modules within eXtensible SRP (XSRP), which allow for selected variants to be implemented. We refer the reader to the project’s repository^2^ for further documentation on the library.

**Figure:**
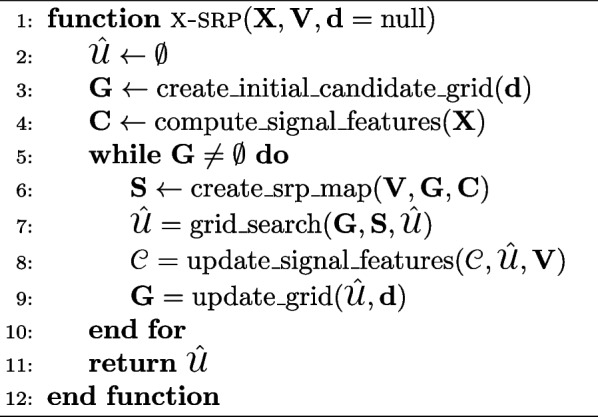
**Algorithm 1** X-SRP

Algorithm 1 accepts three input parameters: a matrix $\textbf{X}$ of microphone signal frames, a matrix of microphone positions $\textbf{V}$ and a vector $\textbf{d}$ containing the room dimensions. It is made optional as it is only necessary for SSL, not for DOA estimation. Note that configuration parameters such as the sampling rate $f_s$ are omitted for the sake of conciseness.

The first line initializes the estimated source coordinates $\hat{\mathcal {U}}$ as an empty set. $\hat{\mathcal {U}}$ is a set of points and not a single point to accommodate multi-source localization approaches.

Then, an initial grid of candidate positions $\textbf{G}$ is created using the **create_initial_candidate_grid** module. In most SRP variants, this will be the only grid created. However, in iterative approaches such as [43, 48] as well as multi-source approaches [28], this function only provides an initial grid $\textbf{G}$, which is further updated as part of their grid search or refinement procedure. Typically, the grid created is a 2D or 3D Cartesian grid for SSL, or a polar or spherical grid for DOA estimation. In the latter case, the room dimensions $\textbf{d}$ are not used, as the grid is produced with respect to the microphone array’s centre. This grid can typically be computed as a pre-processing step if the microphone and room geometries are known beforehand.

The **compute_signal_features** module computes $\textbf{C}$, which can be the CC function between microphone pairs, their GCC-PHAT, or neural-based features [108, 116].

Line (5) begins a loop, which represents the grid search procedure. For most approaches, this loop will only execute once and will only execute multiple times in approaches such as [43, 48]. We define as the loop’s stopping criterion the candidate grid $\textbf{G}$ being empty, symbolizing that end of the grid search.

The module **create_srp_map** computes the SRP map $\textbf{S}$, which assigns a likelihood value to each grid point in $\textbf{G}$, using the microphone positions $\textbf{V}$ and the temporal features $\textbf{C}$.

Then, the **grid_search** module searches for the grid points in $\textbf{G}$ that maximize the SRP map $\textbf{S}$ to estimate the source coordinates $\hat{\mathcal {U}}$, as well as a new grid of candidate locations $\textbf{G}$. When localizing a single source, grid_search returns $\hat{\mathcal {P}} = \{\text {arg max}_{\textbf{G}} \textbf{S}\}$, and an empty grid, i.e. $\textbf{G} = \emptyset$.

The **update_signal_features** module is used to alter the signal features $\textbf{C}$. This is mainly used in iterative and multi-source approaches such as the source de-emphasis procedure [28] (cf. Fig. 8). Finally, the **update_grid** module may be used to generate a new grid based on the current source estimates $\hat{\mathcal {U}}$. An example of variant using this grid is the SRC approach [48].

## Conclusion

In this paper, we showed that the SRP method remains an important localization method and is still under continuous improvement. We hope that the detailed description of the conventional SRP method, followed by a presentation of the combination of the literature into multiple categories, has allowed the reader to learn or increase their knowledge on SRP. Finally, we hope that the alternative division of SRP into functional blocks will allow for the method to be further expanded.

Future research directions on SRP include further improvement of neural methods, by allowing an arbitrary number of sources to be concurrently detected, inclusion of prior information such as noise statistics as a secondary network input, or architectural modifications, for example. Signal processing-based SRP modifications can also be improved by exploring other types of multi-source and tracking strategies, as well as alternative strategies for combining pairwise and frequency-wise information.

## Data Availability

Code used for generating figures and simulations used on this paper is available in https://github.com/egrinstein/xsrp.

## Footnotes

1 https://github.com/egrinstein/xsrp

2 https://github.com/egrinstein/xsrp
